# Innovative approaches to pericardiocentesis training: a comparative study of 3D-printed and virtual reality simulation models

**DOI:** 10.1186/s41077-025-00348-0

**Published:** 2025-04-04

**Authors:** Alberto Rubio-López, Rodrigo García-Carmona, Laura Zarandieta-Román, Alejandro Rubio-Navas, Ángel González-Pinto, Pablo Cardinal-Fernández

**Affiliations:** 1 Intensive Care Unit, Hospital Universitario HM Montepríncipe, HM Hospitales, Madrid, Spain; 2 Facultad de Medicina, Universidad San Pablo-CEU, CEU Universities, Madrid, Spain; 3 Departamento de Tecnologías de La Información, Escuela Politécnica Superior, Universidad San Pablo-CEU, CEU Universities, Madrid, Spain; 4 Universidad San Pablo-CEU, Madrid, Spain; 5 Universidad Autónoma, Madrid, Spain; 6 Cardiac Surgery Unit, Hospital General Universitario Gregorio Marañón, Madrid, Spain; 7 Cardiac Surgery Unit, Hospital Universitario HM Montepríncipe, HM Hospitales, Madrid, Spain; 8 Intensive Care Unit, Hospital Universitario HM Torrelodones, HM Hospitales, Madrid, Spain; 9 Facultad HM de Ciencias de La Salud de La Universidad Camilo José Cela, Madrid, Spain

**Keywords:** Pericardiocentesis, Medical simulation, 3D-printed mannequin, Virtual reality training, Cognitive load, Heart rate variability, Stress analysis, Resource-limited settings, Hybrid training models, Medical education

## Abstract

**Background:**

Training in invasive procedures like pericardiocentesis is a critical component of medical education but poses significant challenges due to its complexity and infrequent clinical application. Pericardiocentesis is an invasive procedure used to remove excess pericardial fluid from the pericardial sac, typically performed to relieve cardiac tamponade. It requires precise anatomical knowledge, ultrasound guidance, and dexterous needle placement to minimize complications. Simulation-based training, particularly with innovative technologies such as 3D printing and virtual reality (VR), offers accessible and cost-effective solutions. This study compared the effectiveness of 3D-printed mannequins and VR simulations in pericardiocentesis training, focusing on learning outcomes, stress responses, and cognitive load.

**Methods:**

Thirty-five final-year medical students participated in this quasi-experimental study, receiving training with both models in separate sessions under the supervision of two experienced instructors. Learning outcomes were evaluated using the objective structured clinical examination (OSCE), while stress responses were assessed via heart rate variability (HRV), a measure of fluctuations in heart rate that reflect stress levels. Perceived cognitive load was measured with the NASA Task Load Index (NASA-TLX). Wilcoxon signed-rank and Friedman tests were used for statistical analysis.

**Results:**

The 3D-printed mannequin outperformed VR in tasks requiring fine motor skills, such as material handling and drainage placement (*Z* = − 2.56, *p* < 0.05; *Z* = − 2.34, *p* < 0.05). VR training, however, was associated with lower mental demand and effort (*Z* = − 2.147, *p* < 0.05; *Z* = − 2.356, *p* < 0.05). Biometric analysis indicated higher stress levels during mannequin-based training (SD1/SD2, chi-square = 14.157, *p* < 0.01), reflecting its closer replication of real-life clinical conditions.

**Conclusions:**

Both 3D-printed mannequins and VR simulations serve as effective tools for pericardiocentesis training, each offering unique advantages. The 3D-printed mannequin supports tactile skill acquisition, while VR enhances cognitive engagement in a low-stress environment. A hybrid approach—beginning with VR and progressing to 3D-printed models—maximizes training outcomes, particularly in resource-limited settings, where affordable simulation tools can improve access to medical education.

**Supplementary Information:**

The online version contains supplementary material available at 10.1186/s41077-025-00348-0.

## Introduction

Pericardiocentesis is an invasive procedure used to drain excess pericardial fluid from the pericardial sac, typically performed to relieve cardiac tamponade. It involves the insertion of a needle into the pericardial space, guided by anatomical landmarks or ultrasound, to aspirate fluid and restore normal cardiac function. Due to its proximity to critical structures such as the myocardium, coronary arteries, and lungs, the procedure requires precise needle angulation and controlled advancement to minimize complications, including cardiac perforation, vascular injury, and pneumothorax. Despite its importance, it is infrequently performed, making skill acquisition through traditional training methods challenging. The Competency-Based Training in Intensive Care Medicine in Europe (CoBaTrICE) initiative has recognized pericardiocentesis as essential competency for critical care specialists, yet there remains a gap in standardized training methodologies [1]. Simulation-based training has emerged as an effective strategy to bridge this gap, offering a controlled environment for skill acquisition without risk to patients [2]. High-fidelity simulators provide realistic training, but their high costs and infrastructure demands limit widespread accessibility [3, 4].

Consequently, alternative simulation methods—such as 3D-printed models and virtual reality (VR) simulations—have gained traction as cost-effective and scalable solutions [5].

3D printing has revolutionized medical training by enabling the development of anatomically accurate models at a fraction of the cost of commercial simulators [6]. These models have been successfully used in various procedural training settings, including vascular access, cardiac surgery, and ultrasound-guided interventions, demonstrating their effectiveness in improving procedural accuracy and motor skill acquisition [7]. Conversely, VR simulation offers an immersive learning environment that enhances cognitive engagement and procedural sequencing, allowing learners to practice complex decision-making in a stress-free setting [8, 9].

Despite their individual benefits, there is limited research comparing the effectiveness of 3D-printed and VR-based models for procedural training [10]. More importantly, few studies have explored integrating these two modalities into a structured training pathway. While 3D-printed models facilitate hands-on skill refinement through tactile feedback, VR simulations support cognitive processing and procedural familiarization [11]. A hybrid approach—beginning with VR for cognitive scaffolding and transitioning to 3D-printed models for hands-on execution—may enhance skill acquisition while mitigating early-stage cognitive overload and stress responses. This progressive model aligns with experiential learning theories, which suggest that training should transition from conceptual understanding to hands-on application.

To address these gaps, a 3D-printed mannequin was developed to provide tactile feedback and hands-on practice, allowing students to refine the fine motor skills essential for performing pericardiocentesis. Meanwhile, the VR simulation was designed to replicate the cognitive components of the procedure in a controlled and immersive environment, making it a suitable training tool for institutions requiring affordable, scalable solutions [12]. Together, these models aimed to overcome the limitations of high-fidelity simulation, offering a practical, low-cost alternative for resource-limited settings.

This study sought to evaluate the feasibility of integrating 3D-printed mannequins and VR simulations into pericardiocentesis training. Specifically, it aimed to analyze how a structured combination of both modalities influences skill acquisition, cognitive load, and stress adaptation.

To assess these variables, the study employed an objective structured clinical examination (OSCE) to evaluate learning outcomes comparing procedural performance in both simulation models [13]; the NASA Task Load Index (NASA-TLX) to measure cognitive load, examining differences in mental effort and workload [14]; and stress adaptation, analyzing heart rate variability (HRV) as a physiological indicator of stress response [15].

By incorporating objective performance metrics and physiological stress markers, this study provides a comprehensive framework for integrating low-cost, high-quality simulation tools into procedural training. The findings may inform future hybrid training models, making pericardiocentesis education more accessible, scalable, and effective—particularly in resource-limited settings.

## Methods

### Study design

This study employed a quasi-experimental crossover design to evaluate the integration of 3D-printed and virtual reality (VR) simulation models in pericardiocentesis training. Due to logistical constraints, random assignment was not feasible; instead, all participants were exposed to both training modalities in consecutive sessions, allowing for within-subject comparisons while minimizing inter-group variability. To ensure methodological consistency, the training sequence was standardized, with participants first completing the VR simulation, followed by a 10-min rest period to restore baseline physiological parameters, and then proceeding with the 3D-printed mannequin. This order was selected to facilitate progressive skill acquisition, enabling participants to develop cognitive frameworks in VR before engaging in hands-on procedural practice.

Both training models were developed using cost-effective materials and open-source software. The 3D-printed mannequin was designed with Tinkercad and Autodesk Fusion 360, printed using UltiMaker Cura and MeshMixer. The VR simulation was created in Unity and delivered via an Oculus Rift S headset, incorporating six degrees of freedom (6DOF) to allow realistic user interaction.

### Participant recruitment and ethical considerations

The study was conducted following approval from the HM University Hospitals Research Ethics Committee (code: 18.12.1339.GHM) and adhered to the Declaration of Helsinki. Participants were recruited from final-year medical students enrolled in advanced procedural simulation courses. To ensure a homogeneous baseline, students with prior experience in pericardiocentesis were excluded. Additional exclusion criteria included the use of medications affecting heart rate variability (HRV) and poor HRV signal quality due to motion artifacts or noise interference. Before training, all students provided written informed consent and completed a standardized instructional module on pericardiocentesis. This module included a theoretical lecture covering the anatomy, indications, and complications of the procedure, followed by a video demonstration to ensure uniform baseline knowledge. All participants completed core clinical rotations in internal medicine, cardiology, and critical care. While most had previous experience with medical simulation, none had performed pericardiocentesis on real patients.

### Development and validation of the training models

Both training models underwent a two-round Delphi validation process, conducted by a panel of five critical care specialists, to assess anatomical and procedural fidelity, usability, realism, and instructional effectiveness. Experts reviewed the 3D-printed mannequin to confirm that thoracic landmarks and pericardial structures provided an accurate reference for needle insertion and catheter advancement, ensuring that the simulated pericardial effusion realistically replicated fluid aspiration mechanics. The VR simulation was evaluated for procedural accuracy and visual realism. Although haptic feedback in VR was limited to controller vibrations, the immersive environment was validated as an effective tool for reinforcing procedural flow and cognitive sequencing.

**Fig. 1:**
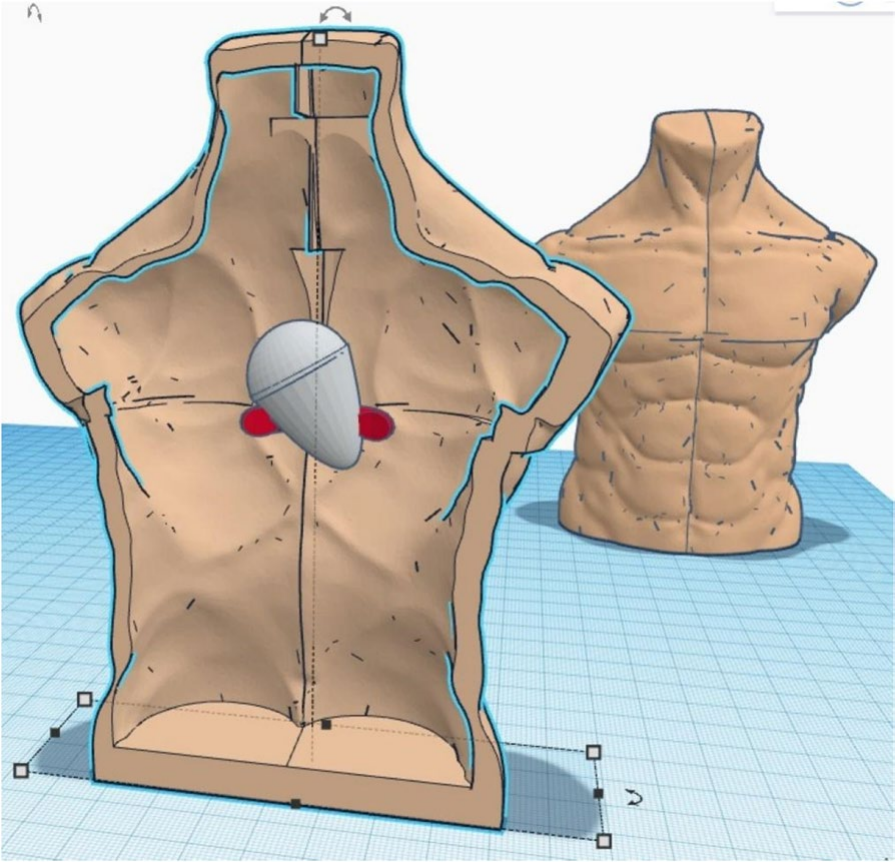
3D Design of mannequin, pericardium, and support, made with Tinkercad

**Fig. 2:**
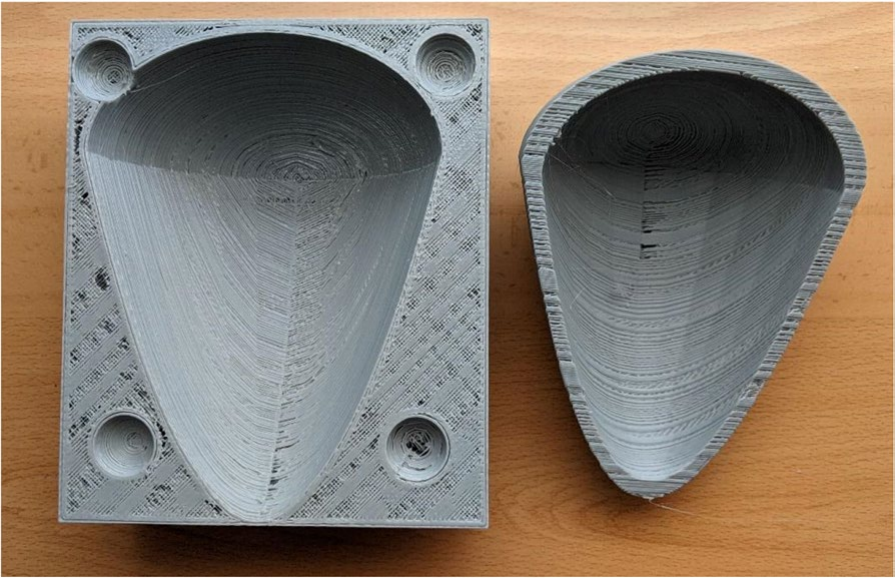
Pericardium 3D-printed mold, laminated with UltiMaker Cura and 3D printed in an Ender-3 printing machine

**Fig. 3:**
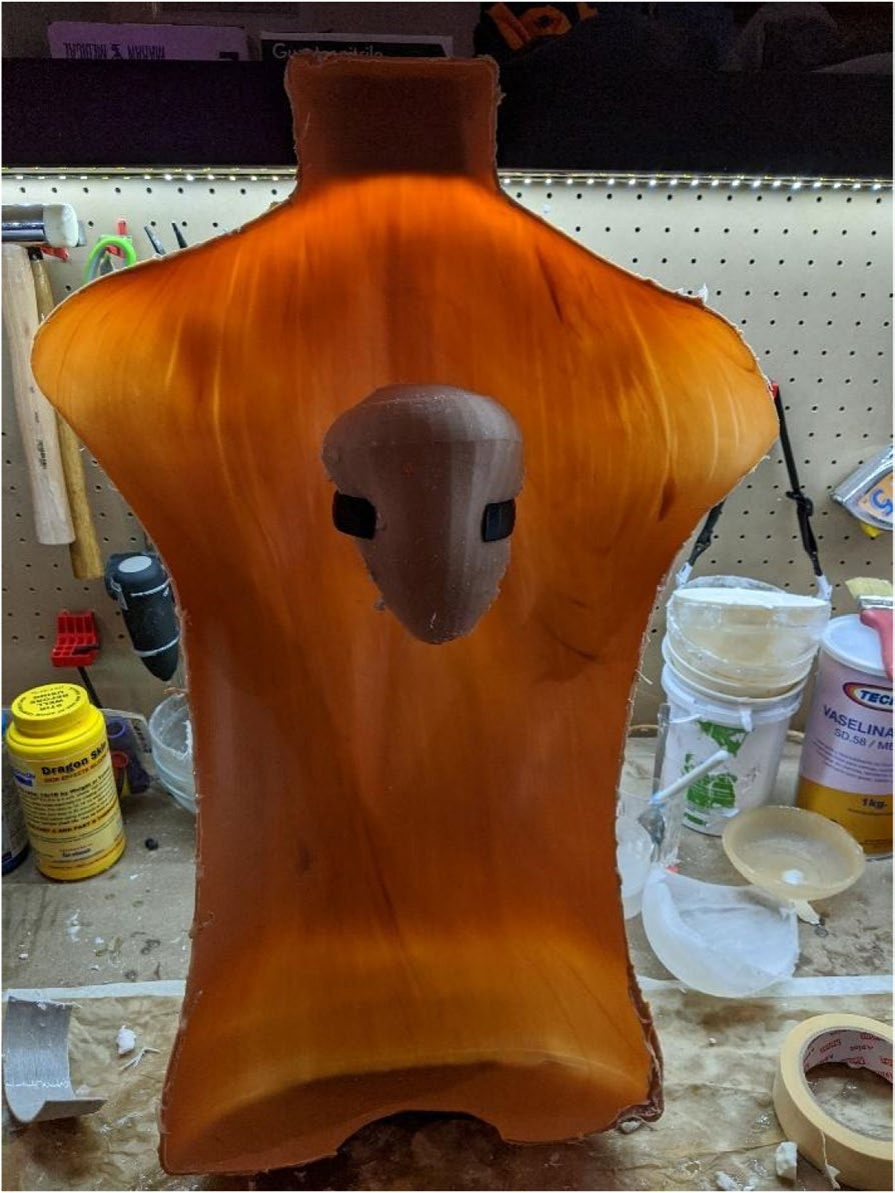
3D-printed pericardium and support

**Fig. 4:**
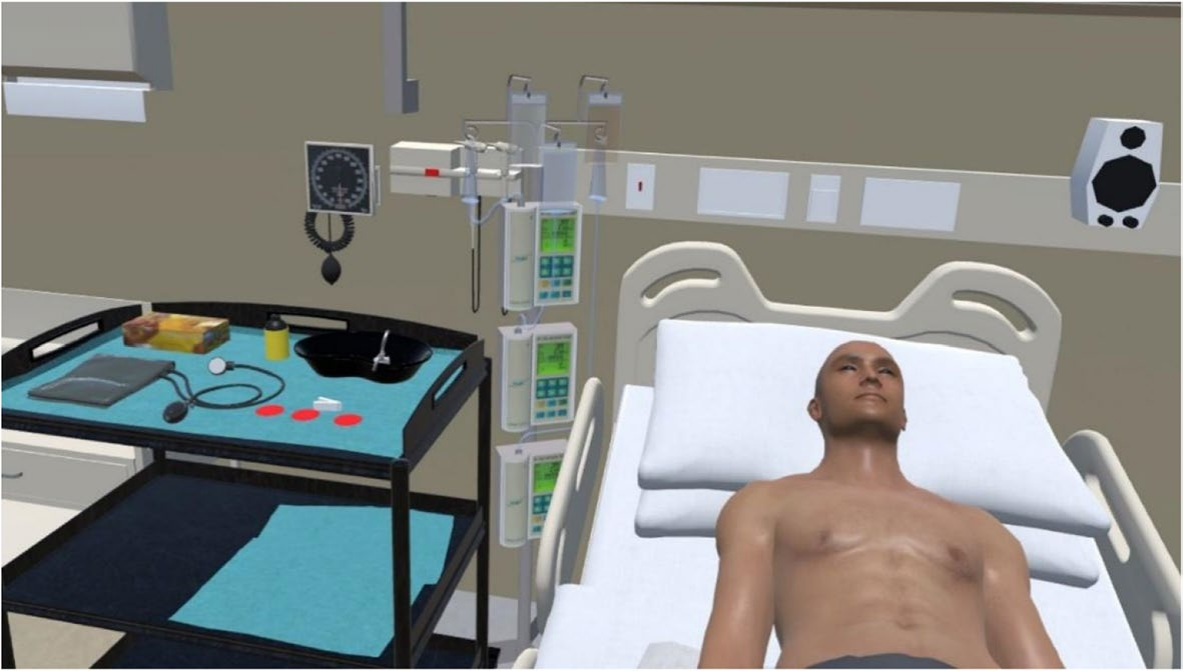
VR scenario still image during pericardiocentesis procedure

- *3D-printed mannequin*: The mannequin was designed to replicate anatomically relevant structures for pericardiocentesis, including the pericardium, thoracic landmarks, and fluid compartments. The model was developed using Tinkercad and Adobe Fusion 360 and printed with open-source tools such as UltiMaker Cura and MeshMixer (Figs. 1, 2 and 3).
- *Virtual reality simulation*: The immersive VR environment, created with Unity, featured six degrees of freedom, enabling realistic user interactions (Fig. 4). Scenarios guided participants through procedural steps with visual and haptic feedback to simulate real-world challenges.

The validation process confirmed that both models were complementary. While the 3D-printed mannequin provided hands-on skill refinement, the VR simulation supported structured cognitive engagement, allowing students to develop a conceptual framework of the procedure before performing manual execution. Minor refinements were incorporated following expert feedback before deploying the models in training.

### Training procedure and data collection

**Fig. 5:**
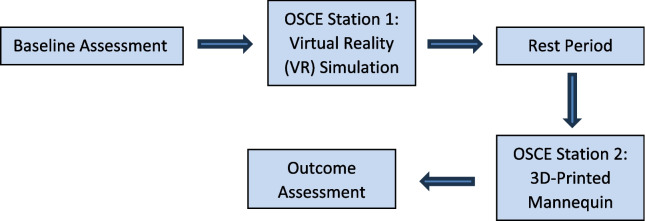
Training procedure

To ensure standardized exposure to both simulation modalities while minimizing potential carryover effects, the study followed a three-phase training protocol (Fig. 5).

1. *Baseline assessment*: Participants provided demographic data and reported prior exposure to medical simulation. A 5-min resting period was conducted to establish baseline HRV parameters.
2. *Training sessions*: Participants first engaged in the VR simulation, followed by a 10-min rest period to restore baseline stress levels, before completing the 3D-printed mannequin session under faculty supervision. This sequence allowed for cognitive familiarization in VR before transitioning to hands-on procedural practice.
3. *Outcome assessment*: Performance was evaluated using a structured objective structured clinical examination, while HRV metrics were recorded throughout the sessions. Cognitive workload was assessed post-session using the NASA Task Load Index.

### Outcome measures

Learning outcomes were assessed using structured OSCE, evaluating aseptic technique, needle angulation, guidewire placement, and overall efficiency. Scores were assigned on a 0–5 scale, with blinded faculty evaluators minimizing assessment bias.

Stress responses were quantified using HRV analysis, with data collected through the Biosignal Plux system and processed in OpenSignals software. The analysis included the following: (1) time-domain metrics: rMSSD, pNN20, and pNN50; (2) frequency-domain parameters: LF, HF, and LF/HF ratio; and (3) nonlinear analysis: SD1/SD2 ratio (primary marker of autonomic stress regulation).

**Fig. 6:**
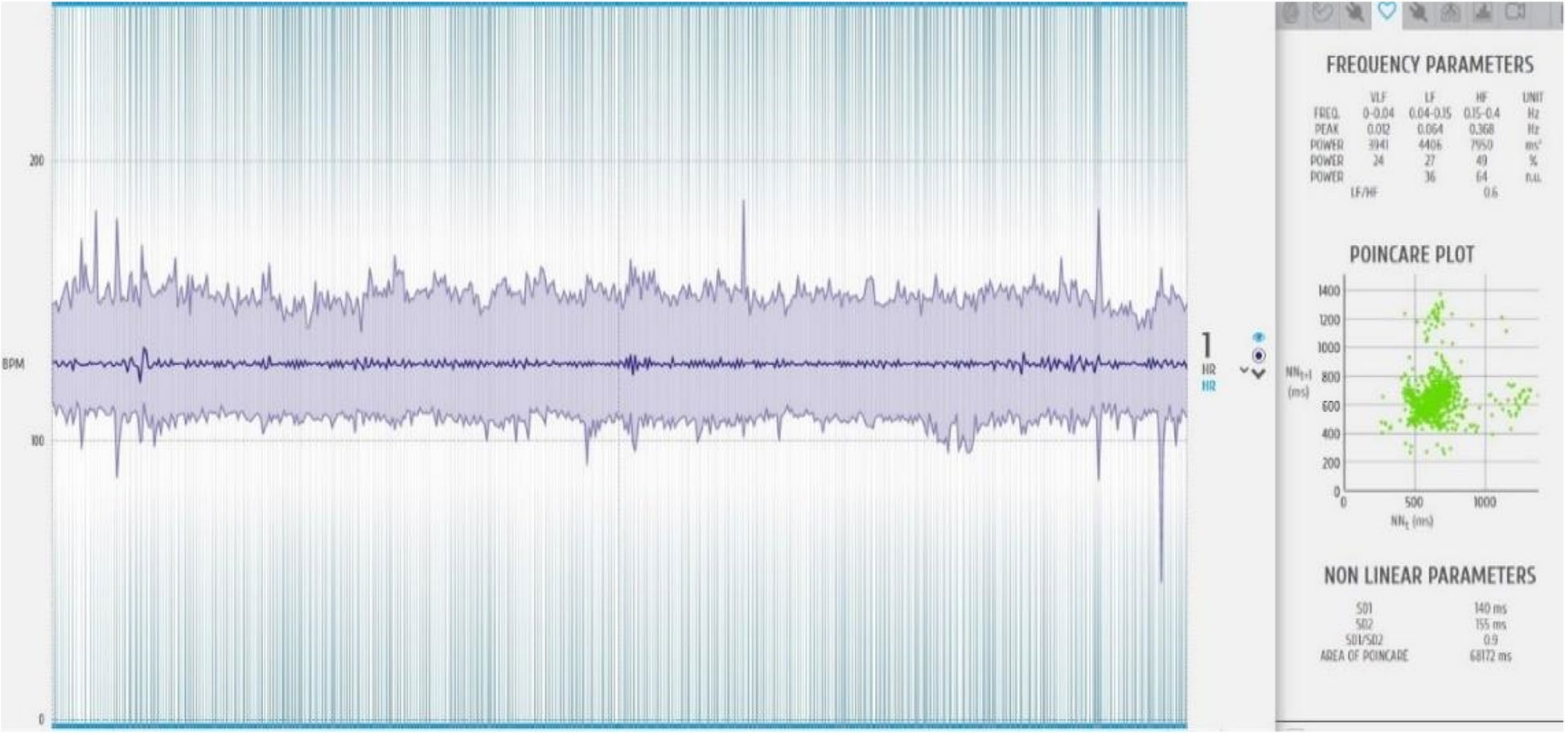
Biosignalsplux digital signal processing tool

To ensure data integrity, signal quality was monitored in real time, and artifacts were removed before processing. Time-stamped recordings ensured synchronization with training phases, allowing for precise comparisons across baseline, VR, and 3D-printed conditions (Fig. 6).

Cognitive workload was assessed using the NASA-TLX, capturing mental demand, physical demand, temporal demand, effort, frustration, and perceived performance. Participants completed the questionnaire immediately after training, allowing for direct comparisons between VR and 3D-printed training experiences.

### Statistical analysis

All statistical analyses were conducted in IBM SPSS version 25. Given the non-normal distribution of the data, nonparametric tests were applied to evaluate differences between training modalities. Wilcoxon signed-rank tests were used to compare OSCE scores and NASA-TLX dimensions, while Friedman tests were employed for within-subject comparisons of HRV parameters across baseline, VR, and mannequin conditions.

Effect sizes (*r*) were calculated for all significant findings following Cohen’s criteria (small: 0.1, medium: 0.3, large: 0.5). To control false positives due to multiple comparisons, Benjamini–Hochberg adjustments were applied, maintaining a false discovery rate of 5%.

## Results

A total of 35 final-year medical students participated in the study, with a mean age of 23.54 years (range: 22–30 years, 65.7% females). Only 8.7% of participants had prior experience with VR-based simulations. No preexisting medical conditions were identified that significantly influenced performance, stress response, or cognitive workload.

### Learning outcomes

Procedural performance was assessed using Wilcoxon signed-rank tests, comparing task execution between the 3D-printed mannequin and VR simulation (Table 1). Participants demonstrated superior performance with the 3D-printed mannequin in tasks requiring tactile feedback and fine motor control, with moderate-to-large effect sizes.

**Table 1:** Comparison of performance between the 3D-printed mannequin and VR simulation across different procedural tasks

Task	*Z*	*p*	*r*
Pulse oximeter placement	− 3.00	0.003	− 0.50
Material handling	− 2.56	0.011	− 0.43
Aseptic technique	− 2.31	0.021	− 0.38
Drainage placement	− 2.34	0.019	− 0.39
Blood pressure monitoring	− 1.11	0.268	− 0.18
Cardiac rhythm analysis	− 1.0	0.316	− 0.17
Needle angulation	− 1.27	0.203	− 0.21

No significant differences were observed in blood pressure monitoring, cardiac rhythm analysis, or needle angulation (*r* < 0.3), suggesting that VR training was sufficient for cognitive aspects but lacked the physical precision required for procedural execution.

### Stress response

**Fig. 7:**
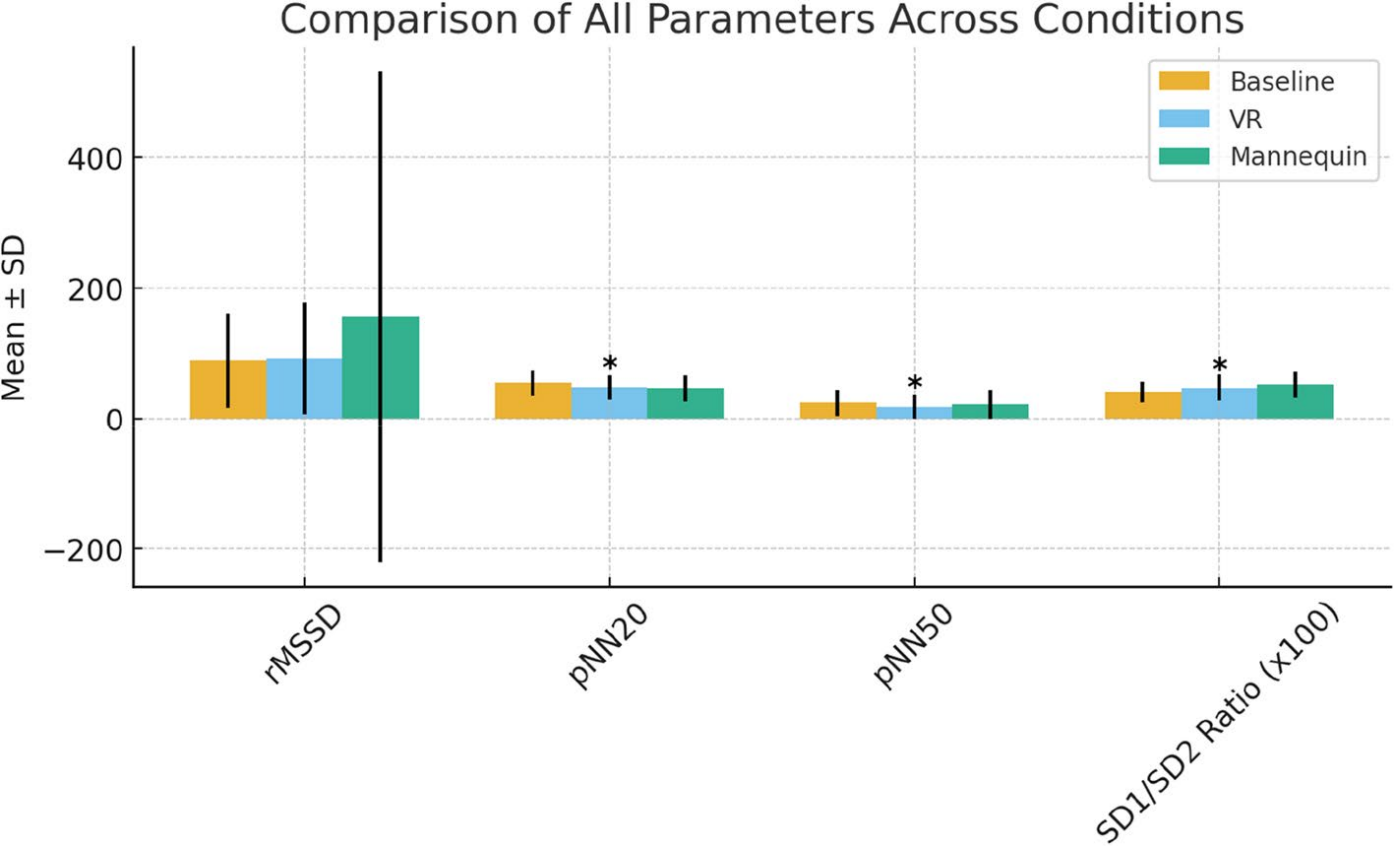
Comparison of heart rate variability (HRV) parameters across conditions

HRV parameters were analyzed using Friedman tests (Table 2), with Benjamini–Hochberg correction applied to account for multiple comparisons. Figure 7 illustrates the differences across baseline, VR, and 3D mannequin conditions. No significant differences were found for low-frequency (LF) or high-frequency (HF) components across conditions (*p* = 0.267 and *p* = 0.229, respectively). However, significant differences were identified in the following:

- *pNN20*: Indicating higher parasympathetic activity at baseline than during VR or mannequin-based training (*χ*.^2^ = 7.393, *p* = 0.025, adjusted *p* = 0.041)
- *pNN50*: Similarly higher at baseline compared to both training conditions (*χ*.^2^ = 9.435, *p* = 0.009, adjusted *p* = 0.017)
- *SD1/SD2 ratio*: Significantly elevated during mannequin-based training compared to VR and baseline (*χ*^2^ = 14.157, *p* = 0.001, adjusted *p* = 0.005), indicating a stronger autonomic stress response

**Table 2:** Heart rate variability (HRV) parameters across training conditions

Parameter	Baseline (mean ± SD)	VR (mean ± SD)	Mannequin (mean ± SD)	Chi-square	*p*	Adjusted p	
rMSSD	88.92 (72.58)	91.81 (85.38)	155.42 (375.80)	2.36	0.267		0.274
pNN20	54.89 (19.37)	47.22 (18.89)	45.78 (20.04)	7.393	0.025		0.041
pNN50	23.75 (20.05)	18.03 (18.89)	21.19 (21.79)	9.435	0.009		0.017
SD1/SD2 ratio	0.407 (0.16)	0.465 (0.20)	0.519 (0.20)	14.157	0.001		0.005

These findings suggest that VR induced a lower physiological stress response compared to the 3D-printed mannequin, which more closely replicated the psychophysiological demands of real-world procedural tasks.

### Cognitive load

**Fig. 8:**
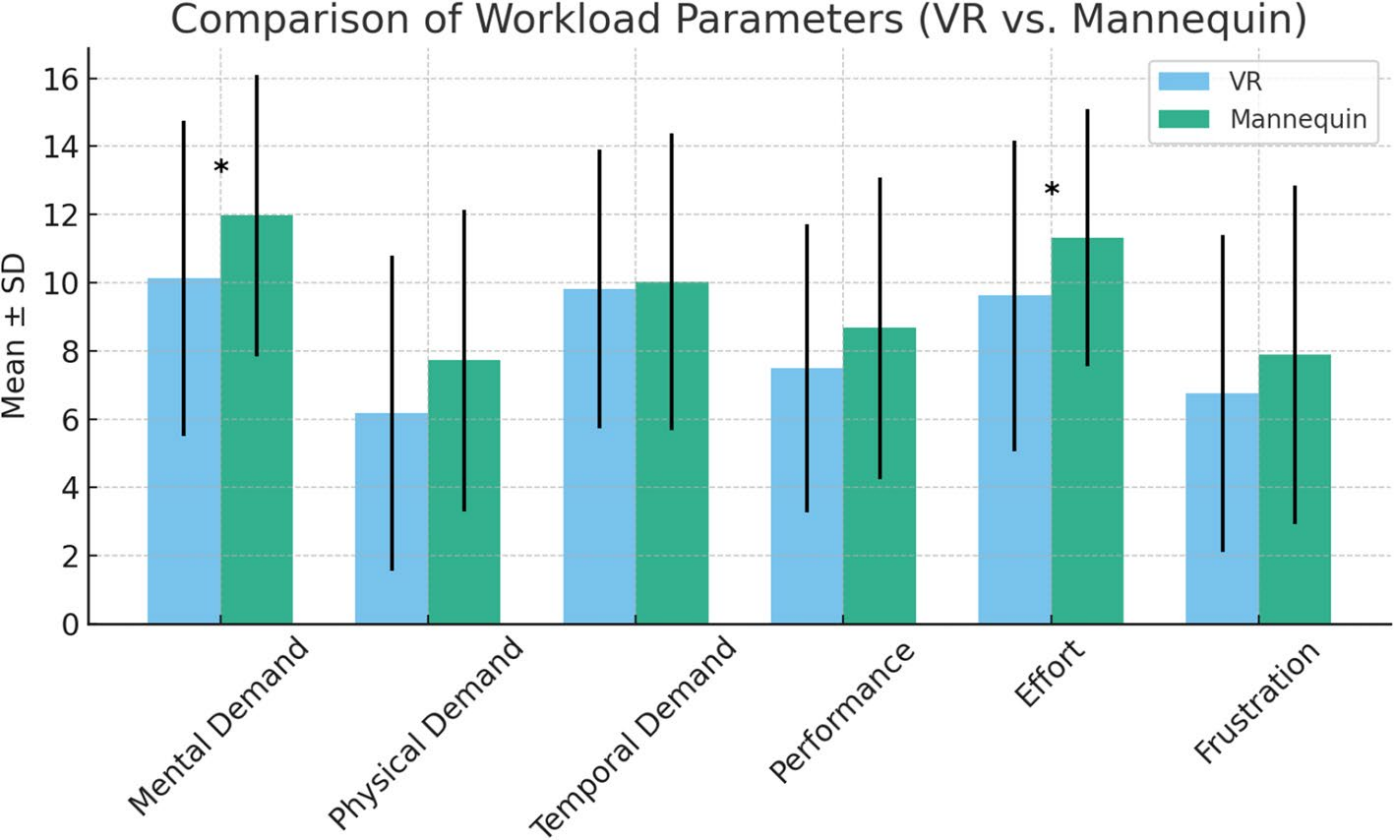
Comparison of cognitive workload parameters between VR and 3D-printed mannequin training

Self-reported cognitive workload scores from the NASA Task Load Index revealed significant differences between VR- and 3D-printed mannequin training (Table 3). Figure 8 illustrates the comparative workload parameters across conditions. Participants reported significantly lower mental demand and effort during VR training:

- Mental demand (*Z* = − 2.147, *p* = 0.032, *r* = − 0.36)
- Effort (*Z* = − 2.356, *p* = 0.018, *r* = − 0.39).

**Table 3:** NASA-TLX cognitive workload scores between training modalities

Parameter	VR (mean ± SD)	Mannequin (mean ± SD)	*Z*	*p*	Effect size (*r*)
Mental demand	10.13 (4.61)	11.97 (4.12)	− 2.147	0.032	− 0.36
Physical demand	6.18 (4.62)	7.72 (4.43)	− 1.263	0.207	− 0.21
Temporal demand	9.82 (4.09)	10.03 (4.36)	− 0.476	0.634	− 0.08
Performance	7.50 (4.22)	8.67 (4.41)	− 1.057	0.290	− 0.17
Effort	9.63 (4.55)	11.31 (3.77)	− 2.356	0.018	− 0.39
Frustration	6.75 (4.64)	7.89 (4.96)	− 0.834	0.404	− 0.14

No significant differences were found for physical demand, temporal demand, performance, or frustration (*p* > 0.05). These findings indicate that VR training imposed a lower cognitive workload, potentially making it more suitable for initial procedural familiarization, while the 3D-printed mannequin required greater cognitive effort, aligning with the demands of hands-on procedural execution.

## Discussion

This study provides a comparative evaluation of 3D-printed mannequins and VR simulations for pericardiocentesis training, emphasizing their complementary strengths. Findings indicate that while the 3D-printed mannequin was superior for tasks requiring fine motor precision and tactile feedback, the VR simulation facilitated early cognitive engagement with the procedural sequence, allowing learners to familiarize themselves with decision-making steps while experiencing lower cognitive load and stress levels. These results align with growing evidence supporting hybrid simulation strategies, in which trainees progress from cognitive familiarization in VR to hands-on skill refinement using physical models. Structuring training in this manner enables a gradual transition from conceptual understanding to procedural execution, optimizing both cognitive efficiency and technical proficiency.

### Hybrid training and learning outcomes

Our results indicate that the 3D-printed mannequin was significantly more effective for procedural tasks requiring fine motor control, particularly material handling, aseptic technique, and drainage placement. These findings align with previous studies demonstrating that tactile feedback enhances motor skill acquisition, a key component of procedural training and psychomotor learning [16].

The use of 3D printing in medical education has been widely recognized as a cost-effective and anatomically accurate alternative to high-fidelity commercial simulators. Prior studies have shown that hands-on engagement with physical models facilitates kinesthetic reinforcement, a crucial mechanism in sensorimotor learning [17]. This advantage has been demonstrated across various procedural contexts, including cardiovascular surgery, ultrasound-guided interventions, and emergency airway management, where 3D-printed models have been shown to improve technical skill proficiency [18].

Conversely, VR-based training was associated with lower cognitive load and effort, allowing participants to engage with the procedural sequence before transitioning to hands-on execution. This aligns with studies indicating that VR supports the development of cognitive schemas, enabling learners to mentally rehearse procedural steps before physical practice [19]. Moreover, VR has been recognized as an effective strategy for cognitive skill acquisition, particularly in resource-constrained environments where access to physical models is limited [20]. Given these complementary strengths, a sequenced training strategy—beginning with VR for procedural familiarization and progressing to hands-on practice with a 3D-printed model—may optimize learning efficiency, balancing conceptual understanding and motor skill refinement while minimizing cognitive overload during early training stages.

### Stress responses and gradual exposure to procedural pressure

Heart rate variability analysis revealed that the 3D-printed mannequin elicited a significantly stronger physiological stress response than VR, as indicated by higher SD1/SD2 ratios. These results suggest that hands-on simulation more accurately replicates the high-pressure conditions of clinical practice, where procedural success is influenced not only by technical skill but also by stress adaptation [21]. This aligns with previous research demonstrating that realistic, hands-on training environments induce autonomic responses comparable to real-life emergencies, reinforcing the role of physical models in procedural stress exposure [22, 23].

In contrast, VR-based training was associated with lower stress levels, likely due to the absence of direct haptic feedback and the controlled nature of virtual environments, which reduce the perceived urgency of the task. While lower stress levels in VR may facilitate early-stage cognitive learning, they may not sufficiently prepare trainees for high-stress clinical conditions. Research on progressive stress inoculation suggests that gradual exposure to stress-enhancing environments improves emotional regulation and decision-making under pressure [24].

A hybrid training model may allow learners to first develop cognitive proficiency in VR, where they can internalize procedural steps in a low-stress setting, before transitioning to mannequin-based training, which introduces haptic complexity and stress adaptation. This structured progression aligns with the stress exposure training framework, which has been widely applied in high-stakes disciplines such as emergency medicine and trauma surgery, demonstrating its effectiveness in building technical competence and stress resilience.

### Cognitive load and learning efficiency

NASA-TLX analysis revealed that participants reported significantly lower mental demand and effort during VR-based training compared to 3D-printed mannequin simulation, while other workload dimensions, such as physical demand, temporal demand, and frustration, remained comparable across modalities. These findings are consistent with studies indicating that VR-based learning environments reduce extraneous cognitive load, allowing learners to process procedural sequences without the immediate burden of fine motor execution [25].

While lower cognitive load in VR may facilitate early procedural comprehension, it may not fully prepare learners for the sensorimotor integration required in real-world execution. Cognitive load theory (CLT) emphasizes that optimal learning occurs when trainees transition from a lower to a higher cognitive demand environment, ensuring that working memory resources are efficiently allocated for skill retention and transfer.

A hybrid curriculum could leverage these findings by using VR to develop cognitive schemas and reinforce procedural steps, followed by hands-on execution with the 3D-printed model, which introduces sensorimotor complexity and higher cognitive demand. This graduated learning approach aligns with progressive difficulty models in simulation-based education, which have been shown to enhance procedural skill acquisition while minimizing cognitive overload [26].

### Implications for training design

The findings of this study hold significant implications for medical education and curriculum development, particularly in institutions with limited access to high-fidelity simulators. Both VR- and 3D-printed mannequins represent cost-effective training alternatives, allowing scalable implementation without the infrastructure and financial constraints of commercial simulators.

To maximize effectiveness, these modalities should be integrated into a structured hybrid training pathway, ensuring a progressive transition from cognitive familiarization to hands-on procedural mastery. A sequenced learning model could begin with VR-based training, where students develop procedural schemas, reduce cognitive load, and practice decision-making in a controlled environment. Following this phase, 3D-printed models could be introduced to enhance kinesthetic reinforcement, fine motor coordination, and stress adaptation, mirroring the transition from theoretical learning to practical execution in clinical training.

This structured learning model aligns with established principles in simulation-based medical education, advocating for gradual skill acquisition, multimodal training, and progressive task difficulty to improve knowledge retention and transferability. By standardizing procedural training through scalable simulation methodologies, medical institutions can enhance skill acquisition, improve patient safety, and better prepare students for real-world clinical challenges.

### Limitations

This study has several limitations. The small sample size may have limited statistical power, particularly in secondary outcome measures. Although significant effects were observed in fine motor performance, stress responses, and cognitive workload, type II errors cannot be ruled out. Future studies should incorporate larger, multicenter cohorts to increase generalizability.

Additionally, the lack of randomization may have introduced selection bias, as all participants underwent both VR- and 3D-printed training in a fixed sequence. Future research should explore randomized controlled trials (RCTs) with counterbalanced sequences to determine the impact of training order on skill acquisition and stress adaptation.

The low familiarity with VR among participants may have influenced both subjective cognitive workload ratings and task performance. Prior studies suggest that VR expertise significantly impacts simulation outcomes, particularly in complex procedural tasks. Future research should examine whether greater VR exposure modifies cognitive load perception and learning efficiency.

Finally, regarding HRV signal processing, while rigorous synchronization and artifact removal techniques were applied, motion artifacts and inter-individual variability remain inherent challenges. Future studies should refine HRV analysis pipelines to enhance data accuracy and reliability.

### Future directions

Future research should focus on assessing the long-term retention of skills acquired through hybrid training, as well as its scalability across different levels of medical education. While this study demonstrated the feasibility of integrating VR- and 3D-printed models in pericardiocentesis training, further investigations should explore how this approach can be adapted for other invasive procedures, including thoracentesis, central venous catheterization, and ultrasound-guided percutaneous interventions. Evaluating the transferability of skills acquired in hybrid simulations to real clinical settings will be essential in determining their effectiveness in improving patient outcomes. Additionally, the continuous evolution of VR technology presents new opportunities to enhance its role in procedural training. The incorporation of high-fidelity haptic feedback, real-time biomechanical modeling, and artificial intelligence-driven adaptive learning could significantly improve VR’s ability to simulate fine motor tasks with greater realism. Research into these advancements could help bridge the current gap between VR-based cognitive learning and hands-on motor skill development, further strengthening the hybrid training paradigm. Finally, future studies should investigate the cost-effectiveness of implementing hybrid training models in different healthcare education systems, particularly in resource-limited settings. Understanding how institutions can integrate these simulations into standardized curricula while maintaining scalability and accessibility will be critical for maximizing their global impact on medical education.

## Conclusions

This study highlights the complementary advantages of integrating 3D-printed mannequins and VR simulations in pericardiocentesis training. While the 3D-printed model proved superior for fine motor skill acquisition, VR facilitated cognitive engagement with lower cognitive load and stress levels, suggesting that each modality addresses distinct yet interdependent aspects of procedural learning. The findings support the implementation of a structured hybrid approach, in which VR serves as an initial cognitive training tool, allowing students to internalize procedural sequences in a low-stress environment, before transitioning to 3D-printed simulation, where they refine motor coordination and stress management under conditions closer to clinical reality. This sequenced progression aligns with best practices in simulation-based medical education, ensuring that trainees develop both conceptual knowledge and hands-on proficiency in a structured manner.

Given its cost-effectiveness and adaptability, this hybrid model has strong potential for integration into medical curricula, particularly in resource-limited settings where access to high-fidelity simulators is restricted. By standardizing procedural training through scalable simulation methodologies, medical institutions can enhance skill acquisition, improve patient safety, and better prepare students for real-world clinical challenges. Future research should focus on long-term skill retention, generalizability across different procedures, and the potential for emerging technologies—such as AI and haptic feedback—to further optimize hybrid training models.

## Supplementary Information

Supplementary Material 1.

## Data Availability

To support reproducibility and facilitate further research, we have made the following resources available: Biometric Data: The anonymized biometric data collected during the study, including heart rate variability (HRV) parameters (e.g., rMSSD, LF/HF ratio), is available through Fighshare at https://doi.org/10.6084/m9.figshare.26215184.v1. 3D Pericardium Model: The 3D model of the pericardium, designed and used for this study, is available for download on https://doi.org/10.6084/m9.figshare.26219135. The model was created using Tinkercad and Adobe Fusion 360 (v. 15.3.0.1657) and can be utilized for educational or research purposes. VR Simulator Repository: The virtual reality simulator developed using Unity 2018.4.36f1 has its source code hosted on GitHub with an open source license, and the application itself can be downloaded freely: https://github.com/rgarciacarmona/VR-Pericardiocentesis. Virtual Simulation Video: A video demonstration of the virtual reality pericardiocentesis simulation, showcasing the immersive environment is hosted on YouTube: https://youtu.be/75eq2mssiRc?si=zlXWpPKFbl4_qwoV. These resources aim to promote transparency, reproducibility, and collaboration within the field of medical education.

## Abbreviations

3D: Three-dimensional
6DOF: Six degrees of freedom
AI: Artificial intelligence
CLT: Cognitive load theory
CoBaTrICE: Competency-based training in intensive care medicine in Europe
HF: High frequency
HRV: Heart rate variability
LF: Low frequency
NASA-TLX: NASA Task Load Index
OSCE: Objective structured clinical examination
pNN20: Percentage of R-R intervals differing by more than 20 ms
pNN50: Percentage of R-R intervals differing by more than 50 ms
RCT: Randomized controlled trial
rMSSD: Root-mean-square of successive differences
SD1/SD2: Standard Deviation Ratio of Poincaré Plot, an indicator of autonomic balance
SET: Stress exposure training
VR: Virtual reality
