# Supplementary material for: Innovative approaches to pericardiocentesis training: a comparative study of 3D-printed and virtual reality simulation models
Source: Adv Simul (Lond). 2025 Apr 4;10:19. doi: 10.1186/s41077-025-00348-0 (PMC11971860; doi:10.1186/s41077-025-00348-0)

**Appendix A**

Links to pericardiocentesis procedure video:

- <https://youtu.be/ox-2LP_3q_k?si=S93Dr3aB27IizWrr>
- <https://youtu.be/OGhQYUfpX2I?si=nvTU0-yv9qGT36YI>.

**Appendix B**

This appendix presents HRV data from one participant in both the mannequin and VR scenarios. HRV, or heart rate variability, reflects the body’s stress response, with lower HRV values typically indicating higher stress levels. In this study, HRV metrics such as the LF/HF ratio and SD1/SD2 were used to assess physiological stress induced by each training model.

**Mannequin scenario**


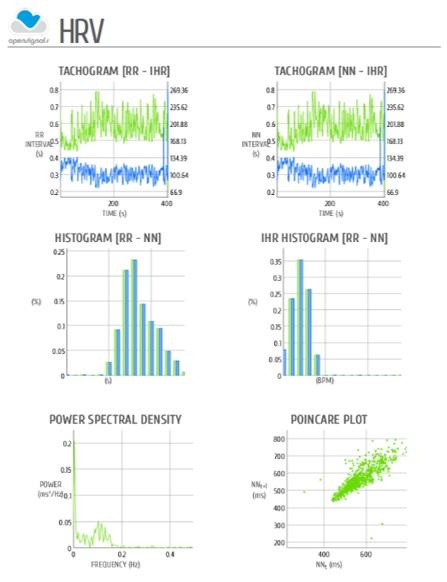


**VR scenario**


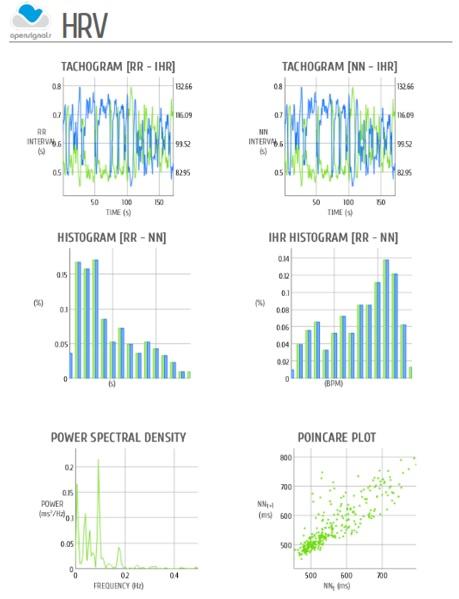

Supplement: Supplementary file 1 — Supplementary Material 1. [file 41077_2025_348_MOESM1_ESM.docx]
